# Disparate participation by gender of conference attendants in scientific discussions

**DOI:** 10.1371/journal.pone.0262639

**Published:** 2022-01-21

**Authors:** Melika Rezaee, Audrey Verde, Benedict Anchang, Sarah A. Mattonen, Jordi Garcia-Diaz, Heike Daldrup-Link

**Affiliations:** 1 Department of Radiology, Stanford University School of Medicine, Palo Alto, California, United States of America; 2 National Institute of Environmental Health Sciences, Biostatistics and Computational Biology Branch, Triangle Park, North Carolina, United States of America; 3 Department of Medical Biophysics, Western University, London, Ontario, Canada; 4 Universtity of Alabama at Birmingham, Birmingham, Alabama, United States of America; Georgia Institute of Technology, UNITED STATES

## Abstract

One important metric of a radiologist’s visibility and influence is their ability to participate in discussion within their community. The goal of our study was to compare the participation level of men and women in scientific discussions at the annual meeting of the Radiological Society of North America (RSNA). Eleven volunteers collected participation data by gender in 59 sessions (286 presentations) at the 2018 RSNA meeting. Data was analyzed using a combination of Chi-squared, paired Wilcoxon signed-rank and T-test. Of all RSNA professional attendees at the RSNA, 68% were men and 32% were women. Of the 2869 presentations listed in the program, 65% were presented by men and 35% were presented by women. Of the 286 presentations in our sample, 177 (61.8%) were presented by men and 109 (38.1%) were presented by women. Of these 286 presentations, 81 (63%) were moderated by men and 47 (37%) were moderated by women. From the audience, 190 male attendees participated in 134 question-and-answer (Q&A) sessions following presentations and 58 female attendees participated in 52 Q&A sessions (P<0.001). Female attendees who did participate in Q&A sessions talked for a significantly shorter period of time (mean 7.14 ± 17.7 seconds, median 0) compared to male attendees (28.7 ± 29.6 seconds, median 16; P<0.001). Overall, our findings demonstrate that women participated less than men in the Q&A sessions at RSNA 2018, and talked for a shorter period of time. The fact that women were outnumbered among their male peers may explain the difference in behavior by gender.

## Introduction

Building a diverse biomedical workforce is essential to excellence in patient care and clinical research [1]. Diverse teams are better at solving complex problems, relate better to the general public, provide improved care for minorities and female patients [2,3] and offer diverse role models for minority trainees [4]. Among the 20 largest residency training specialties, diagnostic radiology ranks 9^th^ in terms of size. However, radiology ranks 17^th^ in terms of women representation with women representing 27% of radiology residents [5]. Women representation is even more scant in leadership positions; with only 26% of radiology faculty being women, and less than 10% of radiology chairs or presidents of private practice groups [6,7].

To achieve professional success, radiologists must be effective and convincing communicators. A large-scale analysis of over eight million scientific papers revealed that men predominate in the first and last authorships of the written scientific communications [8]. However, little information is available about the participation of men and women radiologists in oral scientific discussions. Participation in scientific discussions may directly and indirectly influence the perceived quality of a researcher and physician [9]. Speaking up at scientific meetings increases visibility and influence within the discipline. Studies from a wide range of fields, including biology, geophysics and genetics, suggest that women under-participate in question-and-answer (Q&A) sessions following presentations [10–13]. However, it is not known, if the same problem applies to the field of Radiology.

Understanding differences of men and women in self-promoting behavior, peer-promoting behavior and participation in scientific discussions could provide a new angle for supporting the recognition, visibility and career success of women radiologists [9]. This could ultimately promote faculty career advancements for women and minorities [9]. In order to evaluate potential gender disparities in oral communications at scientific meetings, we evaluated the level of participation of female and male attendees in Q&A sessions following presentations at the annual meeting of the Radiological Society of North America (RSNA). Based on the results, we generated recommendations to optimize women’s participation in scientific discussions.

## Materials and method

### Data source

This study did not require Institutional Review Board (IRB) approval as it does not meet the definition of human subject research defined in 45 CFR 46 nor the FDA definition of a clinical investigation as defined in 21 CFR 56.

Eleven faculty and trainees volunteered to collect data at the 2018 RSNA meeting. One researcher reviewed the RSNA 2018 program to identify the presenter’s and moderator’s gender for all of the presentations listed in the program. Volunteers used Google search engine to find each presenter’s professional profile (provider profile and/or university profile). If the gender was not listed on the profile, then photographs were used to assign gender based on gender presentation. For the minority of presenters for whom no professional profile was found, gender was assigned based on name etymology. Each scientific session included multiple individual presentations. The volunteers attended scientific sessions based on their professional interest. Each volunteer was provided with a template spreadsheet to collect the following data for each presentation they attended: presentation title, presenter’s gender, number of male and female moderators in the panel, number of men and women from the audience who asked question(s) during the Q&A session and speaking time for each gender. Volunteers classified the gender of participants based on stereotypical western gender expressions including physical appearance such as dress, hair, make-up, body language and voice. Participants who expressed their gender with more traditionally masculine or feminine traits were classified as a man or woman, respectively.

We defined audience participation as speaking during the Q&A session following each presentation by asking a question(s) and/or commenting on the presentation’s topic. We excluded the speaking time of the moderators or the speakers during the Q&A session. The participation level of attendees from the audience was measured by the total number and time (seconds) each gender spent at the microphone asking a question and/or commenting during the Q&A session. Volunteers used the *GenderAvenger [14]* application to count and record the speaking time for male and female participants during the Q&A session. All participants in the Q&A sessions were included; these were radiologists, radiology trainees, medical students and scientists with an age of 18 or older. Data were collected from 286 presentations in 59 sessions at the RSNA meeting. Data were collected from presentations that covered various topics such as health policy and quality improvement, education, gastrointestinal imaging, interventional radiology, breast imaging, neuroradiology, chest imaging, nuclear medicine, informatics and artificial intelligence, genitourinary and kidney imaging, physics, pediatrics and molecular imaging.

### Statistical analysis

#### Conference-sample gender ratio participation

Chi-squared analysis was used to compare the gender ratio of speakers and attendees’ participation during the Q&A sessions in our study sample to the gender ratio of speakers and attendees in the entire RSNA meeting, respectively.

#### Question participation analysis across sessions

To further account for differences in gender participation across all Q&A sessions, we performed a non-parametric paired Wilcoxon signed-rank test to compare the total number of men and women who participated in the Q&A session following the presentation associated with each session. A value of P<0.05 was considered significant.

#### Time participation analysis

We next focused on the independent Q&A session following each presentation in which men and/or women from the audience spoke at the microphone. We used a paired t-test to investigate the difference in paired total speaking time in seconds for male and female participants from the audience during the Q&A session after each presentation. Our hypothesis was that there would be no difference in mean speaking time between male and female participants. A value of P<0.05 was considered significant.

## Results

According to information provided to us by the RSNA registry, 32% of all professional attendees were women and 68% were men. Similarly, of the 2,869 presentations listed in the 2018 RSNA program, 35% were presented by women and 65% were presented by men. We evaluated 59 out of 549 total RSNA sessions for our assessment. These 59 sessions comprised 286 presentations, of which 109 (38.1%) were presented by women and 177 (61.8%) were presented by men. There was no statistically significant difference between the presenter’s gender ratio in our selected 286 presentations and the overall presentations (2869) in the meeting (P = 0.3). These 59 sessions (286 presentations) were moderated by a total of 47 (37%) women and 81 (63%) men (Table 1).

**Table 1:** Summary of gender ratio in the 2018 RSNA meeting and our selected 286 presentations.

**Entire 2018 RSNA Meeting**	**Women**	**Men**
Attendants	32%`	68%
Presenters	35%	65%
**Our Study- 286 Presentations (59 sessions)**		
Moderators	37%	63%
Presenters	38%	61%

In our sample, we observed a significantly higher number of men compared to women who asked questions in the Q&A sessions (P<0.01). Women represented 32% of conference attendees and participated in only 24% of the Q&A sessions. These proportions differ significantly (P<0.01).

**Fig 1:**
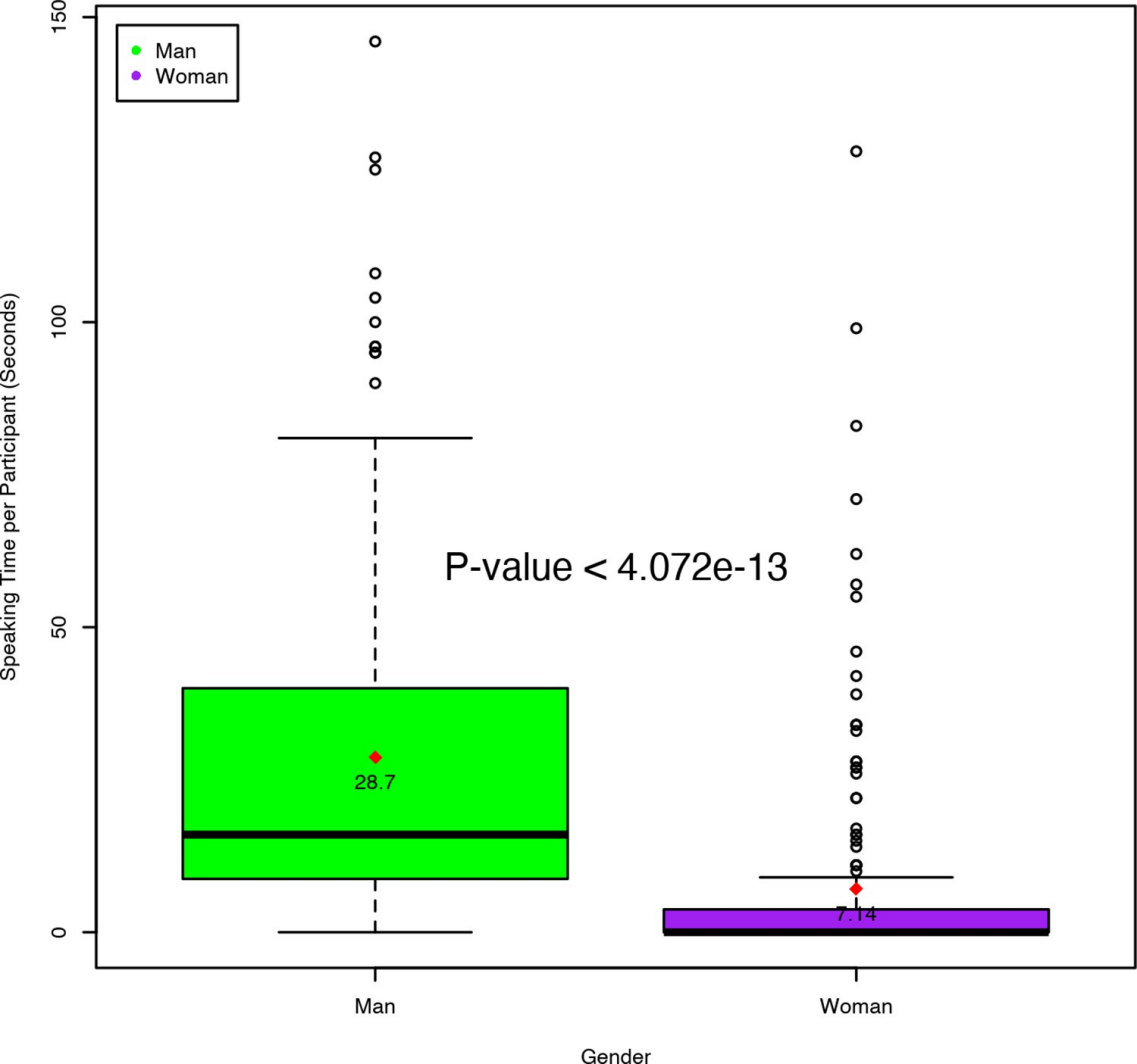
Speaking time. Box plots showing the median and average speaking time per male and female participants from the audience by gender.

The overall speaking time per audience participant was greater for men than for women. Speaking times ranged from 4–128 seconds for female participants and 3–146 seconds for male participants. It was notable that men often started with a general introduction followed by their question, while most women directly asked their question without much introduction. The mean speaking time for women during Q&A sessions (7.14 ± 17.7 seconds) was 4.0 times shorter compared to the mean speaking time for men (28.7 ± 29.6 seconds). This difference was statistically significant (p< 0.001) (Fig 1).

## Discussion

Our study highlights gender disparities in the participation of male and female radiologists and scientists in scientific discussions at a major Radiology conference. In our study sample, women in the audience participated in 24% of Q&A sessions even though they represented 32% of conference attendees. In addition, women who participated in the Q&A sessions, talked for a significantly shorter period of time compared to men.

Poor visibility at scientific meetings has been identified as one of the barriers to women’s academic success [3]. Participation in scientific discussions can help build professional reputations and advance research ideas and thereby, support the participant’s career development [3]. Prior studies document that women are underrepresented at major scientific meetings, both in number and in participation level [9,13,15]. This decreased number in attendance could contribute to their under-participation in scientific discussions and decreased visibility on a national and international stage. Within the literature, and the daily lived experience of women, it is repeatedly demonstrated that women who self-promote and communicate their work publicly in male-dominated fields often face negative implicit bias [16] and stereotypes such as being labeled “bitchy”, seen as lacking “credibility”, or judged purely on “appearance” [17]. Due to these daily challenges, women may feel reluctant to speak up in male-dominated environments, and even more so if the male colleagues are older or more senior in the academic/private hierarchy.

In addition to societal expectations creating a sense of imposter syndrome impacting participation, it is possible that participation is also influenced by the behavior of panelists and moderators. For instance, panelists and moderators may unconsciously support male participants asking questions in male-dominated fields. Some of our study participants reported that women were asked by panelists to “keep their comments short” or “be concise” due to time constraints. Future studies should investigate if such comments are made more often to women than men during discussion sessions. Moving forward, it would be helpful if speakers and moderators could make a conscious effort to encourage all attendants to participate and engage in discussions, and equally enforce time limitations. Moazzam et al. observed that women were more likely to ask questions if another woman asked the first question. Indeed, they suggest moderators to deliberately give the first question to a woman during Q&A sessions [18]. This is another strategy that moderators can employ to best ensure equal gender representation during Q&A participation. Gender difference in active participation at academic meetings is also seen in other fields outside of medicine. Telis et al. evaluated the participation of women at academic meetings for Human Genetics and found that women asked fewer questions and that this gender gap in participation exceeded disparities in their underrepresentation. They further examined the effects of specific interventions to improve this gender gap and found that public discussion of women under-participation alters question asking behavior. Once this disparity was highlighted in a plenary talk on the opening night of the annual meeting, the proportion of talks with zero questions from women decreased substantially from 51% to 30% [9]. Perhaps this strategy can be employed at future RSNAs.

In the field of radiology, women are underrepresented in advanced academic ranks and leadership positions. Increasing the diversity of our workforce will allow us to better serve our diverse patient populations, provide a broader range of perspectives to clinical care and research questions, and increase the number of female role models [2,19] ultimately inspiring a more diverse group of medical students to join this incredible field. Our study shows that including women in our field is more nuanced than enabling both men and women to sit in a lecture room, because not everyone has equal power or visibility. Often, even in rooms that seem gender-diverse, men still dominate the conversations. Although few initiatives have increased diversity in the field of radiology, actively discussing the need for all participants to join the conversation will ultimately lead to a broader exchange of ideas among all participants [20].

Our study had some limitations. Data was limited to 286 presentations in one conference. In the future, we plan to recruit more volunteers to collect data from more sessions. The volunteers attended and collected data from sessions based on their professional interest; even though volunteers were recruited from all subspecialties in radiology, the lack of a systematic randomization procedure had potentially influenced the arbitrary selection of sessions for this study. In addition, we did not collect the total number of men and women attendants in the audience of each session. Collecting information about the total number of attendants for each session is challenging since many walk in or out in the middle of a session. Therefore, the number of attendants per session is difficult to quantify.

The gender of presenters, moderators and participants was classified based on their professional profiles, their name’s etymology or gender expression which inevitability introduces bias. Volunteers assigned gender on the assumption that the presenter’s gender expression represented their internal gender identity, which will not always be an accurate assumption. Future studies will also focus on accounting for the observed differences in participation levels for different gender interaction groups across various sessions across multiple conferences.

We hope our study will increase awareness of how participants of different genders engaged in discussion during RSNA 2018 and ignite a conversation of how we can all work together to make our field more diverse and inclusive. Being an active participant at conferences is a rewarding opportunity to share insights and ideas, and to build a network across our large community. As a field, there are many ways we can better support our women and underrepresented members so that success and advancement are equally achievable. The results from our study can serve as a baseline measure of the gender representation and participation gap of professional attendees at RSNA, and can encourage interventions to increase the participation of women in future radiology meetings.

## Supporting information

S1 File (CSV)
